# Comparison between Rolled and Nonrolled U-Shaped Flap in the Second Stage of Dental Implant Surgery: A Randomized Clinical Trial

**DOI:** 10.1155/2022/1329468

**Published:** 2022-10-04

**Authors:** Ahmed Fadhel Al-Quisi, Osamah Mohammed Aldaghir, Hassanien A. AL-jumaily

**Affiliations:** ^1^Oral and Maxillofacial Surgery Department, College of Dentistry, University of Baghdad, Baghdad, Iraq; ^2^Oral and Maxillofacial Surgery Department, College of Dentistry, Al-Muthanna University, Samawah, Iraq

## Abstract

**Background:**

The presence of black triangles around the dental implant-supported prosthesis and the failure to construct adequate papillae around them bothers dental implantologists. Peri-implant surgical soft tissue management will improve esthetics, function, and implant survival.

**Aim:**

To compare the effects of rolled and nonrolled U-shaped flaps combined with a temporary crown in enhancing the soft tissue around dental implants.

**Materials and Methods:**

Forty patients were included in this study; all patients were operated on by the same maxillofacial surgeon at Al-Iraq specialized dental clinics from January 2019 to January 2020. Patients were divided randomly into two groups: group A: at the second stage of implant surgery, a U-shaped flap without rolling was used in conjunction with temporary crown placement; group B: at the second stage of implant surgery, a U-shaped flap with rolling was used in conjunction with temporary crown placement. Then, the temporary crown was fabricated for both groups and kept in place for one month. Two independent maxillofacial surgeons evaluated all patients two weeks after the cementation of the final zirconia crown for the implant soft tissue esthetic score.

**Results:**

The highest possible score assigned to the mesial papilla (2 scores) was present in 92.5% of the group A patients and only 77.5% of the group B patients. Moreover, we have found that alveolar bone contour is achieved perfectly (2 scores) in 70% of group A patients but only in 32.5% of group B patients.

**Conclusion:**

The U-shaped flap without rolling with a temporary crown is a simple technique. It has good results, especially when there is no severe resorption of the labial bone plate (in canine and premolar areas).

## 1. Introduction

Bone resorption and soft tissue changes at the extraction site will occur despite all the measures that may be taken to prevent these changes [1].

The amount of the resultant alveolar bone resorption and the changes in the gingival biotype after tooth extraction depend on the thickness of the labial bone plate. A thin labial bone plate results in more alveolar bone loss with thickening of the mucosa overlying this bone plate [2].

Huynh et al. showed that the thickness of the labial bone plate in the esthetic zone is less than 1 mm in most patients, with resultant severe bone and soft tissue changes after dental extraction [3].

Peri-implant surgical soft tissue management is intended to improve the esthetic results, masticatory function, and implant survival by avoiding food impaction and better hygiene around the dental implant [4–7].

Accordingly, surgical soft tissue management around dental implants has become essential and complementary to the primary surgical phase to achieve better esthetic results.

Thus, replacing a missing tooth in the esthetic zone with a dental implant is highly technique-sensitive. High esthetic results in this zone are multifactorial; thus, osseointegration around these implants may not be enough [8].

Different types of flap designs (U-shaped flap, apically repositioned partial thickness flap, rotated double-pedicle flap, and modified roll flap) are utilized in the second stage of dental implant surgery; each method claims to be superior to the other methods [9–11].

Nemcovsky, Moses, and Artzi (2000) describe using a U-shaped flap in the second stage of the dental implant to enhance papillae formation around dental implants, which involves making two divergent buccal incisions while preserving the adjacent papillae. These two incisions are connected by a palatally placed crestal incision [12].

On the other hand, Abrams (1980) describes a modified roll flap to correct horizontal soft tissue defects. In 2010, Hu et al. utilized this flap to camouflage peri-implant horizontal soft tissue defects and enhance papillae formation. It involves two vertical buccal incisions connected by a palatally placed crestal incision to create a U-shaped flap. Then the buccal incisions are extended to include the adjacent papillae of the adjacent teeth. After that, de-epithelialization of the U-shaped flap was done, followed by folding of the de-epithelialized part buccally and fixing it with a suture [13].

However, the literature did not discuss the combined effects of temporary crowns and flaps made at the second stage of implant surgery for soft tissue enhancement, elimination of black triangles, and papillae construction.

### 1.1. The Aim of the Study

To compare rolled and nonrolled U-shaped flaps in combination with a temporary crown to enhance the soft tissue around dental implants.

## 2. Materials and Methods

Forty patients were included in this study; the same maxillofacial surgeon operated on all patients from January 2019 to January 2020. This study was carried out according to ethical principles and in compliance with the Declaration of Helsinki.

All patients who complained of a single missing tooth in the esthetic zone (upper centrals, laterals, canines, and premolars) with adjacent natural sound teeth were included in this study. All patients with uncontrolled systemic diseases, bad oral hygiene, a history of irradiated jaws, and patients with severe vertical or horizontal bone resorption (checked by cone-beam computed tomography) (CBCT) were excluded from the study.

After fully informed written consent was obtained from patients included in this study, the initial surgical process of the dental implant fixture installation (Endosseous dental implant (tioLogic® DENTAURUM, Germany), size 3.7 mm in diameter with 11 mm and 13 mm in length) was carried out. This surgical process involved the infiltration of local anesthetic solution (Lidocaine 20 mg/ml with epinephrine 0.0125 mg/ml, Normon, S.A. Spain), then measuring the vertical gingival thickness at the alveolar crest with a sterile endodontic file size of 40 with an elastic stopper; after cutting the file tip with a turbine bur to make it blunt. A paracrestal incision was made for an envelope flap with a no. 15c scalpel blade and reflected with a Haworth's periosteal elevator. The dental implant site preparation proceeded with an 800 rpm speed, 35 N·cm torque, and copious irrigation by normal saline. The diameter of the implant kit drills increased gradually into the dimensions of the implant fixture; the depth of the drilling was determined according to the vertical gingival thickness (for example, if the vertical gingival thickness was 2 mm, the fixture was placed at 1 mm subcrestally to achieve the proper emergence profile).

After four months of the initial surgical procedure, patients were divided randomly into two groups by using a permuted block of randomization with Microsoft Excel (2013) to avoid the imbalance of the number of participants in two groups: group A: at the second stage of implant surgery, a U-shaped flap without rolling was used in conjunction with temporary crown placement.

Group B: at the second stage of implant surgery, a U-shaped flap with rolling was used in conjunction with temporary crown placement.

The second stage of surgery was accomplished by infiltrating the local anesthetic solution (Lidocaine 20 mg/ml with epinephrine 0.0125 mg/ml, Normon, S.A. Spain) labially and palatally, followed by making two vertical parallel incisions with a no. 15c scalpel blade on the scalpel handle no. 3. These two vertical incisions were made on the occlusal surface and extended slightly palatal beyond the position of the dental implant fixture without involving the papillae of the adjacent natural teeth. Another horizontal incision was made crestally to connect the vertical incisions [8].

**Figure 1:**
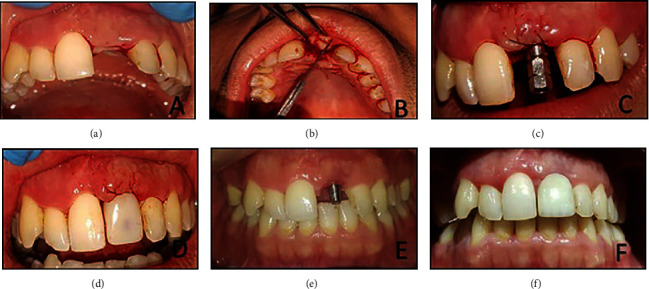
(a) After four months of dental implant installation (note the labial bony resorption) (b) U-shaped flap raised and de-epithelized (c) rolling the flap and suturing it (d) chair side temporary crown (e) after one month (note the camouflage effect of the flap for the labial bone resorption) (f) one month after insertion of the implant-supported permanent zirconia crown.

Reflection of the full-thickness mucoperiosteal flap with Haworth's periosteal elevator was achieved. Afterward, a U-shaped flap was reflected buccally with the placement of the dental implant abutment to construct the temporary crown. In group B patients, an additional step involved de-epithelialization of the occlusal part of the flap with scalpel blade no. 15c. Subsequently, rolling off and fixation of the de-epithelized part of the flap labially with two stitches of 4/0 black silk suture on the mesial and distal sides of the flap [9, 12, 13] (Figure 1).

**Figure 2:**
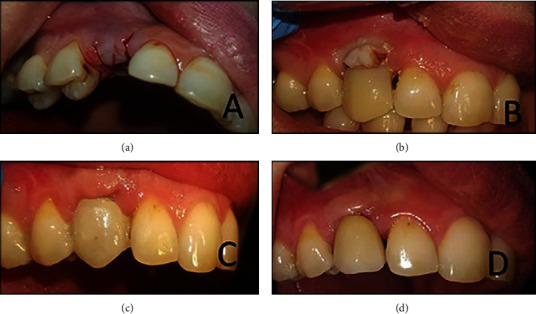
(a) U-shaped flap raised and reflected; (b) chairside temporary crown applied; (c) healing after one month; and (d) final Zirconia crown in place.

The temporary crown was fabricated by using the dental implant abutment with packable and flowable composite. The pressure applied by the temporary crown causes slight blanching of the buccal soft tissue. This blanching should not cross the midline of the adjacent teeth, and it will disappear after half an hour from the application of the temporary crown. If not, then slight trimming of the cervical portion of the temporary crown should be done [14] (Figure 2).

The temporary crown is carefully designed by increasing the subgingival crown convexity and creating a contact line with the adjacent crowns instead of the contact point.

Furthermore, after ten days, further adjustment of the temporary crown until the desired result of the soft tissue around the dental implant in about one month is obtained with the temporary crown.

All patients were evaluated two weeks after the cementation of the final zirconia crown (according to the implant soft tissue esthetic score by Fürhauser et al.) [15].

The evaluation was made by two independent maxillofacial surgeons blinded to the type of flap used with the temporary crown in the second stage of implant surgery. Intraexaminer calibration for each group to check the effectiveness and accuracy of this evaluation method has been done.

The sample size was calculated by using GPower 3.1 software, and the calculation data were obtained from a study by Barakat et al.[8].

The statistical analysis was achieved using the package for social sciences program (SPSS) version 24.0 (SPSS Inc, Chicago, IL, USA). A Shapiro–Wilk test was performed to evaluate the normality of the data distribution. An independent *t*-test was used to evaluate the differences between the independent, normally distributed parametric data regarding patients' mean age. At the same time, the Mann–Whitney *U* test was implemented to evaluate the differences in the abnormally distributed data regarding vertical gingival thickness and the mean value of the implant soft tissue esthetic scores between the two groups. The probability value was considered significant when it was less than 0.05.

## 3. Results

Forty patients (16 males and 14 females) aged between 29 and 45 years old enrolled in this study.

All patients had a vertical gingival thickness of ≥1.5 mm but less than 2 mm (Table 1).

All patients complained of a single missing tooth in the esthetic zone; the distribution of the dental implants according to the position was almost the same for both groups (Table 2).

**Figure 3:**
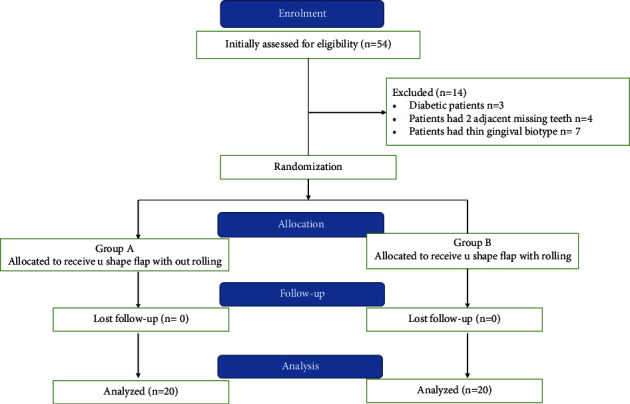
Flow chart of the study.

None of the patients missed the follow-up period (Figure 3).

The two independent maxillofacial surgeons evaluated patients according to the implant soft tissue esthetic score. The results of intraexaminer calibration showed statistically insignificant differences between the scores recorded by both examiners.

The highest possible score assigned to the mesial papilla (2 scores) is present in 92.5% of the group A patients, while it is achieved in only 77.5% of the group B patients.

The distal papilla's highest scores were present in 82.5% of the group A patients, with the same highest score being achieved in only 60% of the group B patients.

Moreover, alveolar bone contour had 2 scores in 70% of group A and 32.5% of group B patients.

When comparing the mean of the scores (from 2 examiners) of the two groups (Table 3), it can be found that the differences between the two groups were statistically not significant. Except for the alveolar bone contour (a highly significant difference between the two groups (*P* value of 0.0071))

## 4. Discussion

Different methods and approaches were tried to get better esthetic and functional soft tissue results around dental implants in the esthetic zone [9–11].

This study presented the advantages of using local flaps and temporary crowns in the second stage of dental implant surgery.

Too many indices were used to evaluate the implant esthetic score [15–17]. In this study, the implant soft tissue esthetic score around a single dental implant was used to evaluate the implant soft tissue esthetic together with the quality of the tissue present around the dental implant that will ensure the health and longevity of the dental implant [15].

Using a U-shaped flap in the second stage of dental implant surgery was introduced by Nemcovsky et al. [12].

The surgical procedure of using a U-shaped flap without rolling was more straightforward and quicker than using a rolled U-shaped flap, which requires de-epithelialization and careful suturing for the rolled flap before placement of the temporary crown. All patients committed to the follow-up visits since their missing teeth were in the esthetic zone, which may explain their adherence to the treatment sessions.

Furthermore, statistical analysis shows statistically insignificant differences between both groups regarding mean scores of mesial papillae, distal papillae, soft tissue contour, color, and zenith line.

These results of the U-shaped flap agree with the findings of Nemcovsky et al.(2000). However, better scores were achieved with the rolling technique, with a statistically significant difference regarding the alveolar bone deficiency of group B patients.

This result can be explained simply by the camouflage action of flap rolling, giving a soft tissue a convexity that makes up for the alveolar bone deficiency.

HuynhBa et al. (2010) showed that the labial bone plate thickness that overlies upper anterior teeth is ≤ 1 mm in 87% of patients [3].

The almost inevitable resorption of the thin labial plate of the alveolar bone after tooth extraction in the anterior maxillary area leads to poor esthetic results in the highly esthetic region of the mouth, which may explain the need for the rolling flap technique in this specific area.

This study showed the importance of customized temporary crowns with these flaps in the second stage of dental implant surgery. Temporary crowns' contact points are placed at a preplanned level (according to the underlying alveolar bone height); temporary crowns play a vital role in shaping dental papillae [18,19].

Within the limitations of this study (short follow-up period, limited number of participants because of the strict selection criteria, absence of gingival biotype measurement),

we can conclude that a U-shaped flap without rolling with a temporary crown is a simple technique and has good results, especially when there is no severe resorption of the labial bone plate. In comparison, the rolling technique can be utilized with greater efficiency in the areas with highly expected bony resorption of their labial bone plates.

## Figures and Tables

**Table 1:** Demographic data of both groups.

Parameters		Group A	Group B	*P* value
Age (mean)		36.05 ± 4.82	36.65 ± 5.20	0.377

Sex	Male	8	6	
	Female	12	14	

Vertical gingival thickness (mean)		1.671 ± 0.121	1.718 ± 0.126	0.312

**Table 2:** Distribution of the dental implants in both groups according to the position

Position of the dental implants	Number of the implants in group A patients	Number of the implants in group B patients
Central incisors	7	8
Lateral incisors	3	4
Canines	2	1
1^st^ premolars	5	6
2^nd^ premolars	3	1
Total number of the dental implants	20	20

**Table 3:** Comparison of the mean scores between the two groups.

Variables	Scores		
	Mean assessment of group A	Mean assessment of group B	*P* value
Mesial papilla	1.75	1.925	0.284
Distal papilla	1.625	1.825	0.28462
Level of soft-tissue margin level (zenith line)	1.75	1.775	0.7948
Soft-tissue contour	1.75	1.7	0.5961
Alveolar process deficiency	1.35	1.85	0.0071^*∗*^
Soft-tissue color	1.95	1.925	0.7948

## Data Availability

The of data used to support the findings of this study are available from the corresponding author upon request.
